# Hyperhomocysteinemia, Suppressed Immunity, and Altered Oxidative Metabolism Caused by Pathogenic Microbes in Atherosclerosis and Dementia

**DOI:** 10.3389/fnagi.2017.00324

**Published:** 2017-10-06

**Authors:** Kilmer S. McCully

**Affiliations:** ^1^Pathology, VA Boston Healthcare System (VHA), Boston, MA, United States; ^2^Pathology, Harvard Medical School, Boston, MA, United States

**Keywords:** allylsulfides, atherosclerosis, dementia, furanonaphthoquinones, homocysteine, thioretinaco ozonide, oxidation

## Abstract

Many pathogenic microorganisms have been demonstrated in atherosclerotic plaques and in cerebral plaques in dementia. Hyperhomocysteinemia, which is a risk factor for atherosclerosis and dementia, is caused by dysregulation of methionine metabolism secondary to deficiency of the allosteric regulator, adenosyl methionine. Deficiency of adenosyl methionine results from increased polyamine biosynthesis by infected host cells, causing increased activity of ornithine decarboxylase, decreased nitric oxide and peroxynitrate formation and impaired immune reactions. The down-regulation of oxidative phosphorylation that is observed in aging and dementia is attributed to deficiency of thioretinaco ozonide oxygen complexed with nicotinamide adenine dinucleotide and phosphate, which catalyzes oxidative phosphorylation. Adenosyl methionine biosynthesis is dependent upon thioretinaco ozonide and adenosine triphosphate (ATP), and the deficiency of adenosyl methionine and impaired immune function in aging are attributed to depletion of thioretinaco ozonide from mitochondrial membranes. Allyl sulfides and furanonaphthoquinones protect against oxidative stress and apoptosis by increasing the endogenous production of hydrogen sulfide and by inhibiting electron transfer to the active site of oxidative phosphorylation. Diallyl trisulfide and napabucasin inhibit the signaling by the signal transducer and activator of transcription 3 (Stat3), potentially enhancing immune function by effects on T helper lymphocytes and promotion of apoptosis. Homocysteine promotes endothelial dysfunction and apoptosis by the unfolded protein response and endoplasmic reticulum stress through activation of the N-methyl D-aspartate (NMDA) receptor, causing oxidative stress, calcium influx, apoptosis and endothelial dysfunction. The prevention of atherosclerosis and dementia may be accomplished by a proposed nutritional metabolic homocysteine-lowering protocol which enhances immunity and corrects the altered oxidative metabolism in atherosclerosis and dementia.

## Infections and the pathogenesis of atherosclerosis

Investigators a century ago considered infectious agents to be critical factors in the pathogenesis of atherosclerosis. The celebrated pathologist and physician William Osler identified the vulnerable plaque as an intimal pustule, and current opinion incriminated infections or toxins as the factor producing intimal lesions of arteries (Osler, 1908). However, efforts to produce arteriosclerotic plaques in experimental animals by infusing bacterial cultures were largely unsuccessful.

Modern techniques of culture, electron microscopy, immunohistochemistry, and molecular hybridization, have demonstrated diverse types of microorganisms within human atherosclerotic plaques. These microbes include *Chlamydia pneumoniae, Helicobacter pylori, Mycoplasma pneumoniae*, archaeal organisms, and more than 50 common pathogenic bacteria (Ott et al., 2006). *Cytomegalovirus* is demonstrated within the smooth muscle cells of human atheromas (Melnick et al., 1983), and biofilms of *Pseudomonas aeruginosa* are demonstrated within atheromas of human carotid arteries by molecular hybridization (Lanter et al., 2014). A diverse population of common bacteria is demonstrated within carotid atheromas by pyrosequencing of bacterial 16s ribosomal ribonucleic acid (rRNA) (Ziganshina et al., 2016). The occurrence of biofilms in human atheromas may explain the failure of anti-microbial therapy to prevent adverse vascular events because of the resistance of biofilms to antibiotics. Experimental atherosclerotic plaques are produced in chickens by Marek's disease herpesvirus (Fabricant et al., 1978). *C. pneumoniae* and *M. pneumoniae* cause exacerbation of arterial plaques in hypercholesterolemic mice (Damy et al., 2009), and *C. pneumoniae* and influenza virus cause exacerbation of arterial plaques in mini-pigs with hypercholesterolemia (Birck et al., 2011).

Since introduction of the cholesterol hypothesis of atherogenesis in 1913 (Anitschkow and Chalatow, 1913) and the homocysteine theory of arteriosclerosis in 1969 (McCully, 1969, 2005, 2016a), evidence has accumulated to support the conclusion that infectious microbes are causal factors in the pathogenesis of atherosclerosis (Ravnskov and McCully, 2012). Mortality from cardiovascular disease increases in infectious diseases like influenza, and other infections predispose to acute coronary syndrome in about one third of cases (Smeeth et al., 2004). Bacteremia and periodontal infections predispose to cardiovascular disease (Valtonen et al., 1993; Spahr et al., 2006), and therapy of periodontal disease improves endothelial function and reduces intimal thickening from proliferation of arterial smooth muscle cells, elastin, and fibrous tissue (Piconi et al., 2009). Septic shock and bacteremia occur frequently in acute myocardial infarction, and serological markers of infection and inflammation are elevated in cases of cardiovascular disease (Espinola-Klein et al., 2002; Ravnskov and McCully, 2009).

Analysis of atherosclerotic plaques by pathologists in the nineteenth century identified inflammation of the arterial wall, deposition of fats in intimal plaques, mucoid degeneration of the media, fibrosis, calcification, and atheroma with crystal deposition as factors in the pathogenesis of atherosclerosis (Virchow, 1856). Following identification of crystals of cholesterol within atheromas (Aschoff, 1924), investigators in Russia produced atherosclerotic lesions in the arteries of rabbits by feeding meat, eggs and milk or by feeding cholesterol to produce fibrolipid arteriosclerotic plaques (Ignatowsky, 1908; Anitschkow and Chalatow, 1913). Investigators in America clarified the role of dietary protein in production of experimental arteriosclerosis by showing that dietary proteins, from which all lipids and cholesterol had been removed by solvent extraction, were responsible for the experimental production of atherosclerosis in animals (Newburgh and Clarkson, 1923).

The contemporary version of the inflammatory pathogenesis of atherosclerosis is the “response to injury” hypothesis, which attributes atherogenesis to factors leading to inflammation, including low-density lipoprotein (LDL), oxidized LDL (oxLDL), hyperhomocysteinemia, hypertension and elevated angiotensin II, and infections by herpes virus and *C. pneumoniae* (Ross, 1999). Elevation of blood homocysteine levels was found to be a pathogenic factor in arteriosclerotic plaques occurring in cases of homocystinuria caused by inherited deficiencies of cystathionine synthase, methionine synthase, or methylenetetrahydrofolate reductase (McCully, 2005). Studies with cultured human macrophages showed that homocysteine thiolactone, the anhydride of homocysteine, causes aggregation and precipitation of LDL and foam cell formation by phagocytosis of homocysteinylated LDL aggregates by cultured macrophages (Naruszewicz et al., 1994). These observations led to development of the homocysteine theory of atherosclerosis and diseases of aging (McCully, 2016a).

Human LDL is demonstrated to be the plasma factor which complexes with microorganisms and their toxins, forming aggregates which inactivate infectious microbes as a critical process in innate immunity (Ravnskov and McCully, 2009). Aggregation and complexation of microbes with LDL are enhanced by hyperhomocysteinemia, because of homocysteinylation of free amino groups of apoB of LDL by homocysteine thiolactone to form homocysteinylated LDL (Naruszewicz et al., 1994). Autoantibodies are formed against homocysteinylated LDL and oxidized LDL, potentially increasing the size of the aggregates of homocysteinylated LDL and microorganisms (Ferguson et al., 1998). The pathogenesis of atherosclerotic vulnerable plaques is attributed to obstruction of vasa vasorum of the arterial wall by these aggregates, causing ischemia, necrosis, hemorrhage, and a micro-abscess, which ruptures into arterial intima, creating the vulnerable plaque (Ravnskov and McCully, 2009). Hyperhomocysteinemia leads to further obstruction of vasa vasorum by microbial LDL aggregates because of narrowing of lumens of arterioles by vasoconstriction, deposition of fibrin, and endothelial dysfunction, and passage of the microbial LDL aggregates through vasa vasorum is further impeded by impaired deformability of erythrocytes and increased blood viscosity (McCully, 1969). Deposition of aggregates of microbes and homocysteinylated LDL within intima and media causes formation of foam cells by phagocytosis of these aggregates by macrophages adjacent to the vasa vasorum, and release of free cholesterol and lipids occurs from degeneration of foam cells within fibrolipid atherosclerotic plaques (Guyton and Klemp, 1993).

## Infections and the pathogenesis of dementia

Extensive evidence supports the conclusion that infectious microorganisms are pathogenic factors in sporadic Alzheimer's disease (Harris and Harris, 2015). Some prominent examples are infections by *Herpes Simplex Virus, Cytomegalovirus*, other *Herpesviridae, Chlamydophilia pneumoniae*, oral spirochetes, *H. pylori, Porphyromonas gingivalis* and other periodontal pathogens and *Propionibacterium acnes*. Six different oral *Treponema* spirochete species were demonstrated in cerebral plaques and intracellular tangles by molecular and immunochemical techniques (Miklossy, 2011). The causative agent of Lyme disease, *Borrelia burgdorfei*, was also detected in cerebral plaques by dark field microscopy, molecular hybridization, and electron microscopy in proven cases of Alzheimer's disease (Miklossy, 2011). Analysis of the historical evidence supports the pathogenic effects of these spirochetes in causing dementia, because of the occurrence of dementia in late stage infections by *Treponema pallidum* or *B. burgdorfei* (Miklossy, 2015). Many of the same pathogenic microbes that are demonstrated in atherosclerotic plaques are also pathogenic factors in the production of Alzheimer's disease.

Deposition of amyloid A-β (Aβ), hyperphosphorylation of tau protein, neurodegeneration, and apoptosis of neurons are reactive processes caused by the presence of pathogenic microorganisms in cerebral senile plaques and intracellular neurofibrillary tangles, according to the microbial theory of the origin of sporadic Alzheimer's dementia (Harris and Harris, 2015). Evidence for this interpretation is the demonstration that Aβ is a polypeptide with anti-microbial properties that is deposited in plaques and tangles as a function of the innate immune system (Socia et al., 2010). Mitochondrial dysfunction is produced by anti-microbial Aβ polypeptides by decreasing fluidity of mitochondrial membranes (Eckert et al., 2001) and opening of the mitochondrial membrane permeability transition pore (Risso et al., 2002), causing release of cytochrome C, induction of breaks in DNA, and cleavage of the poly-ADP ribose polymerase (PARP), the NAD^+^-dependent enzyme that catalyzes repair of DNA (Aarbiou et al., 2006).

## Hyperhomocysteinemia and altered oxidative metabolism in atherosclerosis and dementia

Hyperhomocysteinemia was demonstrated to be a risk factor for dementia of the Alzheimer type by a study of 1,092 participants in the Framingham Heart Study (Seshadri et al., 2002). Participants with hyperhomocysteinemia have an increased risk of subsequent development of dementia after 8 years of observation. Hyperhomocysteinemia is also associated with aging and atherosclerosis. Blood levels of homocysteine increase ~1 μmol/L per decade over the age of 60, and hyperhomocysteinemia is an independent and potent risk factor for development of cardiovascular, cerebrovascular, and peripheral vascular disease (McCully, 2016a). Lowering blood homocysteine levels by B vitamin therapy with folate, cobalamin, and pyridoxal fails to reduce adverse vascular events in subjects with established cardiovascular or cerebrovascular disease. A recent study, however, demonstrates that B vitamin therapy reduces cerebral atrophy in gray matter regions of brain in subjects with cognitive decline who are vulnerable to the Alzheimer's disease process (Douad et al., 2013).

Reductions of brain glucose metabolism and blood flow in the brain of subjects with Alzheimer's disease are demonstrated by positron emission spectroscopy (Chandrasekaran et al., 1996). Synaptic loss or synaptic dysfunction reflect down-regulation of gene expression for glucose transport, Na,K-ATPase, oxidative phosphorylation, and energy consumption in affected regions of the brain. In aging of otherwise normal subjects, mitochondria become progressively dysfunctional because of decreased oxidative phosphorylation resulting from decreased transfer of electrons to the site of oxidative phosphorylation. These changes produce a reduction in oxygen consumption, decrease in mitochondrial membrane potential, increased oxidation products of proteins, DNA, and phospholipids, and increased size, fragility, and bizarre shape of mitochondria in aging animals (Navarro and Boveris, 2007).

## Discovery of thioretinamide and thioretinaco ozonide function in oxidative metabolism

Failure of oxidation of the sulfur atom of homocysteine thiolactone was demonstrated in cultured malignant cells, inhibiting the conversion of the sulfur atom of homocysteine to sulfate (McCully, 1976). The resulting accumulation of intracellular homocysteine thiolactone is attributed to depletion of a hypothetical derivative of homocysteine thiolactone from malignant cells (McCully, 1976). Because of reaction of intracellular homocysteine thiolactone with free amino groups of macromolecules to form peptide-bound homocysteine, the homocysteinylation of proteins, DNA, RNA, glycosaminoglycans, and other macromolecules produces the cellular changes that are characteristic of malignant cells. These changes include increased negative charge of cellular membranes, diffuse membrane dysfunction, increased immunological reactivity to homocysteinylated antigens, irregular aggregation of chromatin, chromosomal abnormalities, altered genetic expression, and mitochondrial dysfunction, especially aerobic glycolysis (McCully, 2016a).

Organic synthesis of derivatives of homocysteine thiolactone disclosed several compounds with anti-neoplastic activity against transplanted malignant neoplasms in mice (McCully, 1992a). The anti-neoplastic activity of these model compounds is associated with solubility in lipids, a carboxyl group adjacent to the nitrogen atom of homocysteine thiolactone, a conjugated double bond system, and complexation with a transition metal ion. The synthetic derivative, thioretinamide (TR), is the amide formed from retinoic acid and homocysteine thiolactone (McCully and Vezeridis, 1987). Thioretinamide forms a complex with the cobalt atom of cobalamin (Co), in which two molecules of TR are bound to cobalamin to form thioretinaco (TR_2_Co) (McCully and Vezeridis, 1989). Both thioretinamide and thioretinaco have anti-neoplastic, anti-carcinogenic, and anti-atherogenic activity in mice and rats, providing evidence for TR_2_Co as the hypothetical derivative of homocysteine thiolactone that facilitates oxidation of the sulfur atom of homocysteine thiolactone and prevents accumulation of homocysteine thiolactone within normal cells (McCully, 2016a).

**Figure 1:**
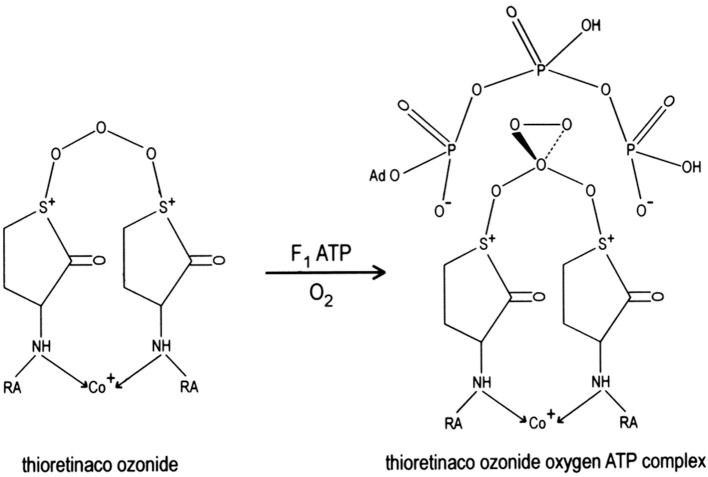
The active site of oxidative phosphorylation is formed by binding of oxygen and ATP to thioretinaco ozonide of the F1 complex of mitochondrial membranes. The alpha and gamma phosphate anions of ATP are bound to the sulfonium cations of thioretinaco ozonide. RA, Retinoic acid; Co^+^, cobalt atom of cobalamin.

Ozone oxidizes the two sulfur atoms of thioretinaco to form a disulfonium complex, thioretinaco ozonide (TR_2_CoO_3_), that binds oxygen and adenosine triphosphate (ATP) to form the active site of oxidative phosphorylation, thioretinaco ozonide oxygen (TR_2_CoO_3_O_2_), within the F1F0 complexes of mitochondrial membranes, as illustrated in Figure 1 (McCully, 1992a). Nicotinamide adenine dinucleotide (NAD^+^) and inorganic phosphate (H_2_${\text{PO}}_{4}^{-}$) form a complex with thioretinaco ozonide oxygen, TR_2_CoO_3_O_2_NAD^+^H_2_${\text{PO}}_{4}^{-}$, which catalyzes ATP synthesis from NAD^+^ and H_2_${\text{PO}}_{4}^{-}$ by reduction of oxygen with electrons from electron transport complexes (McCully, 2015). Reduction of oxygen releases ATP from binding to the active site and produces hydroperoxide radicals which are converted to hydroperoxides by protonation, creating a proton gradient and the mitochondrial membrane potential (McCully, 1992a).

The sulfating coenzyme phosphoadenosine phosphosulfate (PAPS) is formed from the active site of oxidative phosphorylation, thioretinaco ozonide oxygen (TR_2_CoO_3_O_2_), which functions as the source of adenosine phosphosulfate (APS) synthesis from NAD^+^ and hydrosulfate (HSO4^−^) by reduction of the complex with electrons from electron transport complexes, releasing APS and producing thioretinaco hydroperoxide (TR_2_CoO_3_O_2_H) upon protonation (McCully, 2016c). Subsequently, APS reacts with guanosine triphosphate (GTP), which is produced from the active site of oxidative phosphorylation, TR_2_CoO_3_O_2_ATP, to phosphorylate APS to PAPS. These proposed reactions for PAPS biosynthesis in atherosclerosis explain the metabolic pathway for formation of PAPS from homocysteine through the intermediate formation of thioretinamide and explain how hyperhomocysteinemia stimulates production of sulfated glycosaminoglycans (GAG), which are essential components of atherosclerotic plaques (McCully, 2016c).

## Infections, hyperhomocysteinemia, polyamine biosynthesis, and suppressed immunity

All of the pathogenic microorganisms that are demonstrated in atherosclerotic arterial plaques and in cerebral plaques and intracellular tangles in the brain in Alzheimer's disease cause increased biosynthesis of the polyamines, spermine, and spermidine, within infected arterial cells and neurons (McCully, 2016b). In normal uninfected host cells, the polyamines function in genetic translation and expression, cellular proliferation and differentiation, and resistance to stress. Viral, bacterial, and protozoal infections increase biosynthesis of polyamines by increasing the activity of ornithine decarboxylase, the rate-limiting enzymatic process in polyamine biosynthesis (McCully, 2016b). In a study of cultured human stem cells infected by *Chlamydia trachomatis*, the infectious agent of trachoma and venereal disease, inhibition of the cellular biosynthesis of nitric oxide (NO) by inducible nitric oxide synthase (iNOS) was demonstrated to occur through up-regulation of ornithine decarboxylase (Abu-Lubad et al., 2014). Thus, infectious microorganisms cause decreased biosynthesis of NO, an essential component of the destruction of microorganisms by immune processes. No similar studies of biosynthesis of polyamines and NO have been reported in cells infected with *C. pneumoniae*, viruses, archaea, spirochetes, periodontal pathogens, or bacteria that occur in atherosclerotic plaques or cerebral plaques.

The polyamines, spermine, and spermidine, are synthesized in normal cells by transfer of aminopropyl groups from adenosyl methionine to the amino group of putrescene (Tabor and Tabor, 1985). This enzymatic reaction is catalyzed by S-adenosyl methionine decarboxylase and by spermidine synthase. Thus, infections by micro-organisms that are implicated in the pathogenesis of atherosclerosis and dementia cause depletion of intracellular adenosyl methionine because of increased biosynthesis of polyamines (McCully, 2016b). A decreased intracellular concentration of adenosyl methionine causes dysregulation of methionine metabolism by decreased allosteric inhibition of methylenetetrahydrofolate reductase (Jencks and Matthews, 1987) and decreased allosteric activation of cystathionine synthase within infected host cells (Finkelstein et al., 1975), causing increased biosynthesis of homocysteine and leading to the hyperhomocysteinemia observed in atherosclerosis (McCully, 2016a) and dementia (Seshadri et al., 2002).

Increased biosynthesis of large quantities of NO is produced by cells infected by a wide variety of microbial pathogens, and the antimicrobial activity of NO is enhanced by formation of peroxynitrite (OONOO^−^) from NO and superoxide (${\text{O}}_{2}^{-}$) (MacMicking et al., 1997). Reactive oxygen intermediates and peroxynitrite are delivered to the phago-lysosomes of neutrophils and macrophages where nitrative stress and formation of nitro-tyrosine aid in destruction of pathogenic microorganisms (Zaki et al., 2005). The processes of cellular immunity utilize these reactive oxygen and nitrogen radicals to mediate the destruction of *M. pneumoniae, C. pneumoniae, Cytomegalovirus, Staphylococcus aureus*, and other pathogenic microorganisms that are implicated in the pathogenesis of atherosclerosis (Ravnskov and McCully, 2012) and Alzheimer's disease (Harris and Harris, 2015).

Nitrite is a precursor of NO because of reduction by the heme co-factor of cystathionine synthase, inhibiting biogenesis of hydrogen sulfide (Gherasim et al., 2014). The kinetics of the formation of nitrite and peroxynitrite by ferrous heme provides evidence for cystathionine synthase as a source of peroxynitrite and NO (Carballal et al., 2016). Thus, the intracellular deficiency of adenosyl methionine occurring in infected cells and in normal aging impairs the function of these mediators in immunity because of decreased allosteric activation of cystathionine synthase by adenosyl methionine (Finkelstein et al., 1975) and decreased biosynthesis of thioretinamide and thioretinaco (McCully, 2011).

## Adenosyl methionine, thioretinaco ozonide, aging, and immunity

Adenosyl methionine is the sulfonium derivative of methionine that catalyzes many essential transmethylation reactions of homocysteine, nucleic acids, neurotransmitters, proteins, and a wide variety of other acceptor molecules. Adenosyl methionine was discovered as the product of the enzymatic reaction of ATP with methionine (Cantoni, 1953). Although decreased formation of adenosyl methionine is observed in many cultured malignant cells and in aging cells and tissues, the activity of adenosyl methionine synthase is normal in most cultured malignant cells and in aging tissues (McCully, 1992b). A marked deficiency of the tissue and blood concentrations of adenosyl methionine is observed in human and animal aging (Baldessarini and Kopin, 1966; Bohuon and Caillard, 1971; Stramentinoli et al., 1977). A deficiency of thioretinaco ozonide may explain the deficient formation of adenosyl methionine in cancer and aging, since thioretinaco ozonide is depleted from cellular organelles in cancer and aging. A proposal for biosynthesis of adenosyl methionine requires thioretinaco ozonide, oxygen, and ATP (McCully, 1992b).

The decline of immune function with aging (Siskind, 1981) is attributed to the declining cellular concentrations of thioretinaco ozonide and adenosyl methionine within lymphocytes, macrophages, polymorphonuclear leukocytes, and dendritic cells (McCully, 1992b). Loss of cellular thioretinaco ozonide from immune cells may explain the exponential increase in susceptibility to microbial infection in atherosclerosis and dementia with aging. Cystathionine synthase catalyzes biosynthesis of thioretinamide from retinoic acid and homocysteine thiolactone, and subsequent complexation of two molecules of thioretinamide with cobalamin yields thioretinaco (McCully, 2011). Thioretinaco is oxidized by ozone to the disulfonium derivative, thioretinaco ozonide, forming the active site of oxidative phosphorylation, which declines during aging (McCully, 2015).

During immune reactions, antibodies produce singlet oxygen, ^1^${\text{O}}_{2}^{*}$, from the production of hydrogen peroxide by oxidation of water, and singlet oxygen destroys antigens that are bound to antibodies (Wentworth et al., 2000). The production of singlet oxygen is believed to involve hydrogen trioxide (H_2_O_3_) as a key intermediate, and ozone is proposed to originate from antibodies during killing of bacteria (Lerner and Eschenmoser, 2003). Ozone destroys hydrogen peroxide (H_2_O_2_) in the peroxone process which kills microorganisms in the purification of water. Other oxidants, such as peroxynitrite, hydrogen peroxide, superoxide, hydrogen trioxide, and hypochlorite, which are produced by antibody catalysis, by macrophages, or by neutrophils are responsible for killing of pathogens by the immune system, but ozone is considered to be the most reactive of these oxidants (Lerner and Eschenmoser, 2003).

Analysis of cholesterol ozonolysis products from human atherosclerotic plaques provides evidence for the production of ozone during atherogenesis (Wentworth et al., 2003). Ozone selectively inhibits the growth of human cultured cancer cells (Sweet et al., 1980). The inhibitory effect of ozone on growth of malignant cells is related to cellular deficiency of thioretinaco, causing decreased formation of thioretinaco ozonide and increased intracellular ozone, leading to apoptosis of malignant cells. Experimental exposure of animals to ozone causes apoptosis of rat hippocampus cells (Rodriguez-Martinez et al., 2016) and apoptosis of lung alveolar cells in mice (Kirichenko et al., 1996; Kosmider et al., 2010). These studies provide evidence that thioretinaco participates in immune reactions by its reaction with ozone to form thioretinaco ozonide.

Those pathogenic microorganisms which participate in the pathogenesis of atherosclerosis and dementia may deplete infected cells of the active site of oxidative phosphorylation, TR_2_CoO_3_O_2_NAD^+^, because of utilization of this complex for oxidative phosphorylation by these pathogens (McCully, 2016b). Depletion of thioretinaco ozonide and increased polyamine biosynthesis in infected cells produces decreased endogenous biosynthesis of adenosyl methionine (McCully, 1992b, 2016b). In addition, depletion of adenosyl methionine caused by increased polyamine biosynthesis in infected cells produces decreased biosynthesis of thioretinamide because of decreased allosteric activation of cystathionine synthase (Finkelstein et al., 1975). Decreased biosynthesis of thioretinamide leads to decreased formation of thioretinaco from cobalamin and thioretinamide, leading to further depletion of the active site of oxidative phosphorylation, TR_2_CoO_3_O_2_NAD^+^, from infected cells (McCully, 2016b). Support for the proposed depletion of thioretinaco ozonide from cellular membranes in aging (McCully, 1992b) is derived from the observation of reduced concentration of the cobalamin coenzymes, methyl-cobalamin and adenosyl-cobalamin, in human brain tissue in aging, autism and schizophrenia (Zhang et al., 2016a).

## Hydrogen sulfide, allyl sulfides, and immunity

Hydrogen sulfide is a potent gaso-transmitter that senses oxygen concentrations in tissues, and the enzymes cystathionine synthase, cystathionase (cystathionine γ-lyase), and 3-mercaptopyruvate sulfotransferase produce hydrogen sulfide from cysteine (Polhemus and Lefer, 2014). Hydrogen sulfide decreases oxidative stress and counteracts experimental ischemia-reperfusion injury, hypertension, and renal failure (Elrod et al., 2007). In an experimental mouse model hydrogen sulfide attenuates neurodegeneration and neurovascular dysfunction induced by intracerebral administration of homocysteine (Kamat et al., 2013). Hydrogen sulfide was found to attenuate myocardial ischemia-reperfusion injury by preservation of oxygen consumption by mitochondria (Elrod et al., 2007). The beneficial vascular effects of the allyl sulfide constituents of garlic, diallyl trisulfide, and diallyl disulfide, are mediated by hydrogen sulfide (Benavides et al., 2007).

The allyl sulfides, diallyl trisulfide, and diallyl disulfide, are responsible for the homocysteine-lowering effect of aged garlic extract that is observed in folate-deficient rats by increasing adenosyl methionine in liver, impairing the remethylation of homocysteine to methionine by inhibiting methylenetetrahydrofolate reductase (Jencks and Matthews, 1987) and by enhancing the conversion of homocysteine to cystathionine by cystathionine synthase (Yeh and Yeh, 2006). In an animal model diallyl trisulfide protects against ethanol-induced oxidative stress and apoptosis by stimulating cystathionine synthase and cystathionase activities, thereby increasing production of hydrogen sulfide by cystathionine γ-lyase (Chen et al., 2016). In diabetic rats diallyl trisulfide protects against the oxidative stress and apoptosis induced by hyperglycemia by stimulating production of hydrogen sulfide derived from cystathionine γ-lyase (Tsai et al., 2015). These studies support the function of hydrogen sulfide in mediating the beneficial effects of diallyl trisulfide, diallyl disulfide, and other allyl sulfides on platelet aggregation, hypertension, elevated blood homocysteine, elevated blood cholesterol, oxidative stress, and apoptosis occurring in cardiovascular disease. Diallyl trisulfide is the most potent diallyl sulfide compound in providing beneficial effects, compared with diallyl disulfide and diallyl sulfide, which are less potent. The unsaturated allyl groups of these polysulfide compounds facilitate their entry through plasma membranes into cells, since the corresponding saturated propyl derivatives have no beneficial cardiovascular effects.

Diallyl trisulfide and other organosulfur compounds of garlic have potent anti-bacterial, anti-fungal, and anti-animal effects, facilitating the beneficial effects against atherosclerosis and Alzheimer's dementia, the pathogenesis of which is promoted by infectious microorganisms (Ravnskov and McCully, 2012; McCully, 2016b). The allyl sulfide components of aged garlic extract enhance innate immunity and inhibit proliferation of viral, fungal, parasitic, protozoan and bacterial pathogens, including *Salmonella, Listeria, Escherichia coli, H. pylori, Mycobacterium tuberculosis*, bacterial film pathogens, *Rhinovirus, Cytomegalovirus, Herpes Simplex Virus, Influenza Virus, Candida albicans, Aspergillus flavus, Cryptosporidium, Toxoplasma, Giardia*, and *Plasmodium* (Reid, 2016). Aged garlic extract modulates innate immunity by increasing the activity of macrophages and natural killer cells and by increasing production of T and B cells, and clinical trials have documented the ability of aged garlic extract to decrease the number, severity, and duration of upper respiratory infections (Reid, 2016).

In a study of nitric oxide (NO) recruitment of monocytes in mice with femoral artery ligation, absence of cystathionine γ-lyase in knockout mice failed to produce NO, but wild type mice with normal cystathionine γ-lyase activity were associated with increased NO production, hydrogen sulfide production and monocyte recruitment in ischemic tissues (Kolluru et al., 2015). Treatment of the cystathionine γ-lyase-deficient knockout mice with diallyl trisulfide restored ischemic vascular remodeling, monocyte recruitment, and cytokine expression by increasing hydrogen sulfide production and restoring NO availability, peroxynitrite formation, and immune destruction of pathogens by reactive oxygen species and reactive nitrogen species delivered to phagosomes of neutrophils and macrophages (Kolluru et al., 2015).

Diallyl disulfide increases mRNA synthesis by the heme oxygenase gene, *HMOX1*, and heme oxygenase is up-regulated in cultured liver cells exposed to ethanol (Charron et al., 2016). Diallyl disulfide also suppresses lactate dehydrogenase and aspartate transaminase activities, suppresses malondialdyhyde concentrations, and increases glutathione concentrations, contributing to protection against ethanol-induced injury to hepatic cells by up-regulation of *HMOX1* (Charron et al., 2016). Thus, allyl sulfides have the capacity to enhance biosynthesis of thioretinamide from retinol and homocysteine thiolactone by oxidizing retinol to retinoic acid through up-regulation of the heme oxygenase function of cystathionine synthase, thereby increasing formation of thioretinaco ozonide and adenosyl methionine and facilitating oxidative phosphorylation in normal and regenerative cells (McCully, 2011).

## Furanonaphthoquinones, napabucasin, diallyl trisulfide, stat3 signaling, and immunity

Screening for synthetic furanonaphthoquinones with cytotoxic activity toward leukemia cells and myeloma cells disclosed the activity of 2-methyl-naptho[2,3-b]furan-4,9 dione (FNQ3) in decreasing growth and increasing apoptosis within cultured human leukemia and myeloma cell lines (Desmond et al., 2005). Normal cell lines are resistant to the cytotoxic effects of FNQ3, requiring a 10-fold increase in concentration to produce apoptosis and growth inhibition. The cytotoxic effect of FNQ3 is mediated by mitochondrial collapse, as measured by depolarization of the mitochondrial membrane, resulting in appearance of a sub-G1 (apoptotic) population of cultured cells. Moreover, FNQ3 markedly enhances granulocytic differentiation of human myeloid leukemia cells in the presence of low concentrations of retinoic acid or dihydroxy-vitamin D3 (Desmond et al., 2005). The mitochondrial dysfunction induced by FNQ3 can be interpreted as competitive inhibition of electron transfer from respiratory complexes to the active site of oxidative phosphorylation, as mediated by Coenzyme Q10, resulting in a decreased proton flow to F1F0 complexes and the resulting decreased membrane potential (McCully, 1992a).

Multiple napththoquinones isolated from the bark and roots of an African shrub, *Newbouldia laevis*, which is used in traditional medicine, were demonstrated to have prominent antifungal and antibacterial properties (Gafner et al., 1996). Additional furanonaphthoquinones, atraric acid and a benzofuran were also isolated from the stem barks of *N. laevis* (Gormann et al., 2003). In a similar study of the roots of the “roble tree” of Puerto Rico and the Dominican Republic, *Ekmanianthe longiflora*, multiple furanonaphthoquinones with antimicrobial and anticancer activity were found to have cytotoxic effects in cultured human breast and lung cancer cell lines (Peraza-Sanches et al., 2000). Studies of naphthoquinones and analogs from *Avicennia* (mangrove) trees disclosed inhibitory effects against mouse skin tumor formation in carcinogenesis testing (Itoigawa et al., 2001). Synthetic derivatives of furanonaphthoquinones isolated from plants were demonstrated to have cytotoxic activity against human tumor cells (Ogawa et al., 2006). The basis for toxicity was considered by the authors to be related to intercalation of furanonaphthoquinones within DNA and free radicals. Two newly discovered cytotoxic naphthoquinones were isolated from the roots and stems of the Madagascar plant, *Mendoncia cowanii* (vahimpianaomby), and were found to be cytotoxic for human ovarian cancer cell lines (Williams et al., 2006). All of these furanonaphthoquinones with cytotoxic activity potentially affect mitochondrial function through inhibition of electron transfer, as mediated by Coenzyme Q10, from respiratory complexes to the active site of oxidative phosphorylation. Malignant cells are more susceptible to the cytotoxic effects of these furanonaphthoquinones because of the lower concentration of thioretinaco ozonide within the mitochondrial membranes of malignant cells, compared with normal cells.

The signal transducer and activator of transcription 3 (Stat3) is frequently detected in breast cancer cell lines but not in normal breast epithelial cells, and a virtual database screening protocol disclosed a natural product molecule, deoxytetrangomycin, an angucycline antibiotic (National Cancer Institute 628869), with potent inhibitory activity against Stat3 activity in human breast cancer cell lines (Song et al., 2005). A systematic study of naphthoquinone derivatives with inhibitory activity against Stat3 signaling in cancer stem cells disclosed a novel class of furanonaphthoquinone molecules with inhibitory activity against cancer stem cell proliferation (Jiang et al., 2014). The most active of these compounds is napabucasin, 2-carboxymethyl-naphtho[2,3-b]furan-4,9-dione, which inhibits gene transcription that is dependent upon Stat3, thereby suppressing gene expression of cancer stemness, blocking spherogenesis of cultured cancer cells, and causing apoptosis of a wide variety of cancer cell types (Li et al., 2015). In a study of human prostate cancer cell cultures, napabucasin was demonstrated to increase apoptosis, inhibit cell proliferation, cell motility, cell survival, and colony formation ability of prostate cancer stem cells (Zhang et al., 2016b). Moreover, an *in vivo* study demonstrated that napabucasin inhibits growth of prostate cancer xenografts from these cancer cell cultures in athymic mice.

Diallyl trisulfide, the cancer chemopreventive constituent of garlic, inhibits phosphorylation of Stat3 in prostate cancer cells in culture and *in vivo* (Chandra-Kuntal and Singh, 2010). In addition, diallyl trisulfide inhibits prostate cancer development in a transgenic mouse model, correlating with a decrease in phosphorylated Stat3. Diallyl trisulfide also inhibits migration of prostate cancer cells, an indicator of metastatic potential, in an *in vitro* membrane migration assay. In a study of mouse colitis induced by dextran sulfate, diallyl trisulfide suppresses Stat3 and NF-κB expression, resulting in decreased inflammation of the colon (Lee et al., 2013). Thus, the inhibitors of Stat3, including organosulfur polysulfides and furanonaphthoquinones, not only have antineoplastic effects, but they are potent antimicrobial and anti-inflammatory molecules with potential benefits in patients with atherosclerosis or dementia.

## Stat3 inhibition and autoimmunity

In patients with germline loss of function mutations of Stat3, immunodeficiency syndromes are observed, and germline gain of function mutations of Stat3 are associated with myelodysplastic syndrome, T-cell large cell granular lymphocytic leukemia, and aplastic anemia (Milner et al., 2015). These gain-of-function *STAT3* mutations confer increased Stat3 transcription, impaired cytokine signaling, and diminished T regulatory cell function. The authors suggest a rationale for use of inhibitors of Stat3 for therapeutic benefit in patients with gain-of-function *STAT3* mutations. In support of this concept, autoimmunity, hypogammaglobulinemia, lymphoproliferation, and mycobacterial disease were demonstrated in patients with activating mutations in *STAT3* (Haapaniemi et al., 2015).

In knockout mice with *STAT3*^−/−^ mutations crossed with CD4+ mice, T helper 17 lymphocytes were found to be absent from intestinal lamina propria (Harris et al., 2007). Experimental autoimmune encephalomyelitis (EAE) requires Stat3 activation, and the *STAT3*^−/−^ CD4 mice were found to be resistant to EAE. Mitigation of disease was also demonstrated in *STAT3*^−/−^ CD4 mice with induced autoimmune pneumonitis. These findings were interpreted to indicate that pathogenic T_*H*_17 cells are dependent upon Stat3 signaling and that inhibition of this signaling pathway results in mitigation of autoimmune disease progression (Harris et al., 2007). The authors suggest that Stat3 inhibition may provide a target for autoimmune diseases. In knockout mice with *p53*^−/−^ mutations crossed with CD45.1 mice, spontaneous autoimmunity was ameliorated by inhibition of the Stat3 signaling pathway and suppression of T_*H*_17 effectors (Zhang et al., 2011). This study identifies a critical function of the tumor suppressor oncogene p53 in suppressing autoimmunity through the Stat3 pathway and T_H_17 differentiation.

## Endothelial dysfunction, endoplasmic reticulum stress, misfolded protein response, and neurodegeneration

Hyperhomocysteinemia promotes endothelial dysfunction, one of the earliest manifestations of atherosclerosis (McCully, 1969, 2009). Homocysteine induces apoptosis of cultured human endothelial cells by activation of the unfolded protein response, which is signaled by the endoplasmic reticulum kinase IRE-1 (Zhang et al., 2001). The unfolded protein response to homocysteine is produced by endoplasmic reticulum stress, which leads to apoptosis in the pathogenesis of human and experimental atherosclerotic plaques (Lhotak et al., 2011). Homocysteine activates Herp, an endoplasmic reticulum protein which facilitates endoplasmic reticulum stress. Herp is expressed in parkinsonian substantia nigra glial cells and is present in the core of Lewy bodies of neurons (Slodzinski et al., 2009). The ozonolysis products resulting from reaction of ozone with cholesterol in atherosclerotic plaques are suggested to produce the protein misfolding of Aβ that is observed in Alzheimer's disease (Zhang et al., 2004).

Cyanobacteria produce the neurotoxic amino acid β-methylamino alanine (BMAA), and the etiology of amyotrophic lateral sclerosis (ALS)/Parkinsonian dementia complex observed in Chamorro residents of Guam who consume fruit bats is traced to consumption of BMAA from cycads containing cyanobacteria (Spencer et al., 1987). In a subsequent study BMAA was detected in the brains and spinal cords of North American patients with sporadic AD and ALS (Pablo et al., 2009). The neurotoxic amino acid BMAA is mis-incorporated into proteins of human cell cultures in place of L-serine, causing protein misfolding and aggregation (Dunlop et al., 2013). The mouse sticky mutation is characterized by follicular dystrophy, hair loss, cerebellar Purkinje cell loss and ataxia, and a mis-sense mutation of the alanyl-tRNA synthase gene was demonstrated to produce low levels of mischarged tRNA molecules, which results in the misfolded protein response, apoptosis and neurodegeneration (Lee et al., 2006). An animal model of AD consists of feeding BMAA to vervets, producing characteristic neurofibrillary tangles and amyloid plaques in the brain (Cox et al., 2016).

Homocysteine is a potent excitatory neurotransmitter which binds to the N-methyl D-aspartate (NMDA) receptor of neurons, causing oxidative stress, cytoplasmic influx of calcium ions, apoptosis and endothelial dysfunction (McCully, 2009). The oxidized derivative of homocysteine, homocysteine sulfinic acid, is also a potent excitatory neurotransmitter which stimulates glucose uptake through the calcium-dependent AMPK-p38MAPK-protein kinase C pathway in muscle cells (Kim et al., 2011). The Aβ_42_ oligomers in cerebral plaques in AD activate the calmodulin-dependent protein kinase kinase CAMKK2-AMPK kinase pathway through phosphorylation of tau protein, and subsequent hyper-activation of CAMKK2 or AMPK by Aβ produces loss of dendritic spines in hippocampal neurons of transgenic mice for human Aβ precursor protein (Mairet-Coello et al., 2013). These findings suggest that reduction of hyperhomocysteinemia by improved nutrition and normalization of methionine metabolism may be beneficial in prevention and treatment of dementia.

## Detection, prevention and treatment of atherosclerosis, and alzheimer's disease

Early detection of subjects at risk for atherosclerosis is accomplished by determination of plasma homocysteine, C-reactive protein, and Framingham risk factors, including smoking, hypertension, family history, and dyslipidemia. Infectious diseases cause dyslipidemia, but the interpretation of dyslipidemia as atherogenic may be misleading, since the altered plasma lipid profile may reflect the pathophysiological response to infection (Gidding et al., 1998; Apostolou et al., 2009). The dyslipidemia observed in young adults is associated with an increased coronary calcium score later in life, and dyslipidemia was suggested to be an early marker of atherosclerosis (Pletcher et al., 2010). Since there is no association between dyslipidemia and the degree of atherosclerosis (Ravnskov and McCully, 2012), a more likely interpretation is that the increased calcium score later in life may have resulted from spontaneously resolved vascular infections, leading to calcified atherosclerotic plaques.

Early detection of subjects at risk for Alzheimer's disease is accomplished by determination of cognitive impairment by abnormal Mini-Mental State Examination (MMSE) scores, computed tomography or magnetic resonance imaging scans of medial temporal lobe thickness, cerebrospinal fluid Aβ_40_, Aβ_42_, or tau protein, plasma homocysteine, plasma C-reactive protein, and ocular biomarkers (Frost et al., 2010; Douad et al., 2013). Identification of pathogenic microbes by positive culture, sero-positivity, or other methods will determine the antibiotic, vaccination, or other anti-microbial treatment strategy (Harris and Harris, 2015).

Treatment of the metabolic abnormalities produced by pathogenic microbes in atherosclerosis or AD, including hyperhomocysteinemia, increased biosynthesis of polyamines, decreased NO biosynthesis, and impaired oxidative metabolism from depletion of thioretinaco ozonide, may be accomplished by a proposed plasma homocysteine-lowering protocol. The protocol consists of thioretinamide, B vitamins, including methyl-cobalamin, methyl-folate, pyridoxal phosphate, and nicotinamide riboside, ascorbate, Coenzyme Q10, adenosyl methionine, menoquinone, cod liver oil, vitamin D3, pancreatic enzymes, and dietary improvement to minimize consumption of processed foods and to provide adequate dietary protein for prevention of subclinical protein energy malnutrition (Ingenbleek and McCully, 2012; McCully, 2016a). Because of their powerful digestive activity against the polysaccharides, proteins, and nucleic acids of biofilm matrix, pancreatic enzymes, including amylase, trypsin, ribonuclease, and deoxyribonuclease, may disperse biofilms in atherosclerotic plaques and cerebral plaques in AD, increasing susceptibility of the pathogens within biofilms of plaques to antibiotic therapy (Allen, 2016). In addition to these measures, meticulous oral hygiene, antibiotic therapy of dental procedures, consumption of dietary monolaurin and other anti-microbial nutrients, consumption of adequate dietary protein, and avoidance of neurotoxins from foods, vaccines, or environmental contaminants may also decrease the progression of mild cognitive impairment to dementia. The efficacy of this proposed protocol requires validation by a properly designed clinical trial.

## Author contributions

KM is responsible for the conception, literature search, authorship, and approval of the final manuscript. No third party sponsored or participated in this manuscript.

### Conflict of interest statement

The author declares that the research was conducted in the absence of any commercial or financial relationships that could be construed as a potential conflict of interest.
